# Kaposi's sarcoma.

**DOI:** 10.1038/bjc.1991.229

**Published:** 1991-07

**Authors:** A. G. Dalgleish


*Br. J. Cancer* (1991), **64**, 3–6 © Macmillan Press Ltd., 1991

GUEST EDITORIAL

### Kaposi's sarcoma

A.G. Dalgleish

*Clinical Research Centre, Watford Road, Harrow, Middlesex HA1 3UJ, UK.*

Kaposi's Sarcoma (KS) remains an enigma over 100 years after it was first described by a Hungarian dermatologist who ensured his place in the hall of fame by changing his name to the exotic 'Kaposi', marrying his Professor's daughter, and describing one of the most enigmatic of all cancers. Indeed there are considerable doubts as to whether or not KS is a true cancer. Atypical and mitotic cells are extremely rare histological findings and the multiple lesions which are such a predominant feature do not really fit the classical understanding of metastases. Nevertheless the patient suffering from advanced KS involving his gastrointestinal and respiratory tracts who is shortly to expire with a massive haemorrhage is hardly likely to be encouraged by the semantic argument that he does not have cancer after all! (Zeigler & Dorfman, 1988).

KS presents in a variety of guises. Until the HIV epidemic, KS was most common in elderly men of Jewish or East European origin where it presented as a multicentric pigmented 'sarcoma' usually appearing on the legs. It is hard to exaggerate the indolence of KS for it responds to minimal doses of radiotherapy or chemotherapy and the experience of the oncologist who treats KS is demonstrated by the fact that he or she usually only treats cosmetically appropriate lesions. Certainly the wait and see approach is compatible with the patient surviving many years and dying of another disease of old age with the KS intact.

An endemic form of KS was recognised in Central and Eastern Africa from the mid 1950's (Templeton, 1973). Disparate clinical features are recognised in a classification describing four main types: Nodular (benign and slow growing), florid (exophytic version of the nodular form), infiltrative (dense with invasion of the dermis and bone) and lymphadermopathic (predominantly in children with rare cutaneous lesions). A systemic subclassification similar to that used in Hodgkin's disease has since been added with A and B indicating absence and presence of systemic symptoms respectively. Even in Africa, marked epidemiological localisation of KS within regions, tribes and amongst individuals strongly suggests a transmissible infectious agent. This does not exclude however a genetic component to disease susceptibility. No obvious factors have been implemented at the time of writing although there are numerous tantalising clues the most important of which will be reviewed here.

Immunosuppression plays an important role in the development of KS which is recognised as a late complication of patients receiving organ transplants and accounts for about 5% of the malignancies in this population (Penn, 1983, 1979). Although there appears to be an incubation time of about 16 months post transplantation it is intriguing that the lesions usually regress when immunosuppression is reduced. The other association with immunosuppression is the appearance in the 1980's of KS in about 40% of AIDS cases. This association hides some interesting clues. Firstly the proportion of KS in homosexuals with AIDS has gradually fallen to about 20% (is this due to safe sex or the reduction in a co-factor, e.g. the use of a vasodilator like amyl nitrate which was common in the gay community in the 1980's) and secondly other groups infected with HIV tend not to get KS, such as haemophiliacs and drug addicts (Rutherford *et al.*, 1990). Initially it was thought that heterosexuals with AIDS did not get KS. However, this is clearly not the case now. HIV seropositivity is associated with the aggressive systemic and rapidly fatal KS in Africa (Whereas most 'classical' KS cases are HIV negative) (Bayley *et al.*, 1985). In Africa, HIV is probably contracted from heterosexual contact, as intensive studies have failed to demonstrate any significant transmission by homosexual, intravenous drug abuse, contaminated blood products or insect bites (Serwadda *et al.*, 1985). Moreover, occasional HIV seropositive prostitutes are beginning to present with KS lesions in Western cities. Nevertheless, even in Africa the male predominance has been noted since the disease was first recognised there.

Received 21 December 1990; and in revised form 22 January 1991.

### Aetiology

### *Genetic*

Early studies indicated a link between KS and HLA.DR5. Further analysis of those reports suggests that the association of HLA.DR5 with Italian or Ashkenzai Jewish descent may explain the apparent association between DR5 and KS (Papasteriades *et al.*, 1984; Pollack *et al.*, 1983*a*,*b*, 1985). In Caucasian populations in which DR5 is less common, no association with KS is seen. In the USA certain HLA antigens (B35, C4, DR1 and DQ1) were found to be more frequent in the KS population compared to controls (Mann *et al.*, 1990). DR14 and DR53 are more frequent and HLA, B8, C5 and DR3 are less frequent in KS patients than in AIDS patients with opportunistic infections, but not with KS. Several studies report other associations between HLA and KS but these vary considerably depending on the populations studied. Described associations include positive links with DR1, DR2 but low frequency DR3 in KS patients.

Overall these studies do suggest that there is a genetic component to KS which probably affects the response to an infectious or other agent.

### Infectious agents

The argument for an infectious aetiology is compelling and includes the aforementioned clustering of cases in Africa (Beral *et al.*, 1990). However, evidence suggests that HIV itself is not the direct cause. KS has been reported in sexually active homosexual patients who have consistently remained HIV sero negative (Friedman-Kien *et al.*, 1990). As mentioned, HIV sero positive homosexuals tend to get KS, but drug addicts do not. Female sexual partners of bisexual men are much more likely to develop KS than the female partners of intravenous drug abusers. A number of large cohort studies have been published describing the associations with the development of KS in AIDS patients. There are some inconsistencies. For example, an association with amyl nitrates was found in some studies (Mathur Wagh, 1985; Haverkos, 1985, 1987; Osmond, 1985) but not in others (Goedert *et al.*, 1986; Polk *et al.*, 1987; Darrow *et al.*, 1987). However there was a strong implicative association with increased numbers of partners, anoreceptive and 'rimming' practices, and previous episodes of other sexually transmitted diseases. The decline in the incidence of KS in the homosexual communities has followed the introduction of safe sex campaigns and are probably related.

Early studies associated Cytomegalovirus (CMV) with KS (Giraldo *et al.*, 1980). However, more stringent studies have failed to confirm this association. Tissues from KS have now been probed for a large number of known viruses including HIV and other retroviruses as well as DNA tumour viruses. No association has been shown. Attempts to isolate a novel virus from KS patients have been unrewarding. Early reports of a viral-like agent turned out to be mycoplasma although it is still under consideration as having a causative role by its discoverers (Lo *et al.*, 1986). Recently however a retroviral like agent has been reported in some KS specimens and a claim been made that is causally associated (Rappersberger, 1990). However, the literature is littered with reports of virus disease associations which have never been substantiated. Meanwhile innovative approaches in the laboratory have shed new light onto the pathogenesis of KS.

### Growth factors

Salahuddin and colleagues working in Gallo's laboratory attempted to overcome the long standing problem of growing and maintaining KS cells in culture *in vitro*. They identified a growth factor in the medium of HTLV-II infected cells which supported the temporary growth of normal vascular endothelial cells, but not fibroblasts (Nakarmura *et al.*, 1988). Interleukin I and tumour necrosis factor stimulated the growth of KS cells transiently and could be distinguished from the HTLV-II derived factor. The cells supported with this factor were shown to be similar to the spindle cells in KS lesions sharing some of the features. They produced factors that supported their own growth (autocrine) and the growth of other cells (paracrine) including umbilical vein endothelium and fibroblasts. The angiogenic activity of these factors were demonstrated by the development of KS like lesions (of mouse origin yet similar to the human lesions) in nude mice following subcutaneous innoculation (Salahuddin *et al.*, 1988). Further studies have shown that KS cells are more susceptible to these factors than normal endothelial cells and that they appear to be in an activated state with increased levels of mRNA for many known growth factors (Ensoli *et al.*, 1989). This could suggest that KS cells are activated by a virus infection or other event which makes them more susceptible to second events such as exposure to growth factors. An anology might be drawn to the way in which HTLV-1 activates T cells and EBV activates B cells making them prone to malignant transformation (Dalgleish & Malkovsky, 1988). The role of HIV in KS patients has hitherto been associated with immunosuppression and was thought to be analogous to the development of KS in immunosuppressed post organ transplant patients. However, transgenic mice containing only the tat (the potent transactivating gene of HIV) mainifest KS like lesions. Intriguingly only male mice are affected and the skin is the only tissue to contain tat mRNA (Vogel *et al.*, 1988). Nevertheless KS occurs in HIV negative individuals and interpretation of these results requires considerable caution.

### Basic biology

Normal angiogenesis generates new blood vessels most evident in wound healing and is a feature of diabetic retinopathy, haemangiomas, rheumatoid arthritis and tumours. Endothelial cells involved in angiogenesis respond to fibroblastic growth factors and heparin like molecules. Negative regulation is important and may in a large part be due to the secretion of transforming growth factor beta (TGF-b) by pericytes and vascular smooth muscle cells as TGF-b is a potent inhibitor of angiogenic factors. The KS factor described by Salahuddin *et al.* (1988) may be an abnormal form of TGF-b which may over-ride the normal inhibitory regulation. It is thus likely that a mixture of positive cytokine signals and the loss of negative or reguatory signals is required for the development of KS.

### Interactions with cells of the immune system

Three major cell types interact with endothelial cells (EC); neutrophils, monocytes and lymphocytes. Neutrophils dynamically interact with EC and cross into the extravascular space. During inflammation neutrophils home to EC's a process involving the expression of adhesive ligands on leukocytes and induced expression of receptors on EC. Many of these have been characterised and are reviewed elsewhere (Harlan, 1985; Pober, 1988). Unlike neutrophils, monocytes do not appear to mediate damage to EC's although they may be able to significantly influence EC cell growth by secreting both positive factors (GM-CSF) and negative ones (α-Interferon).

Lymphocytes readily emigrate to sites of inflammation and leave the blood by adhering to and migrating through the endothelium lining specialised venules, particularly, though not exclusively, the morphologically distinct high-endothelial venules (HEV). The characteristics of which may depend upon the presence of certain lymphocytes themselves! (Streeter *et al.*, 1988). In addition to leukocytes homing in on EC's and influencing their growth and function (which may include fatally damaging them), ECs may significantly influence leukocytes. For instance, numerous factors and cytokines may activate ECs which will then express MHC class II molecules which will present antigen to T helper cells, and readily induce an allogenic response (Pober, 1988).

The interactions between cells of the immune system and ECs are clearly complex and different facts have been used to describe EC changes in inflammation, vasculitis and atherosclerosis. So which features may be more pertinent to the pathology of KS?

KS cells appear to be of endothelial origin and are consistent with activated EC cells. Indeed human monokines and cytokines from HIV negative patients with induce endothelial cell elongation which resembles the KS spindle cell. Moreover, activation is clearly lymphokine dependent (Groenwegen *et al.*, 1985; Fitzgerald *et al.*, 1987; Majewski *et al.*, 1987; Bussolino *et al.*, 1989). Other cells apart from lymphocytes can secrete EC inhibitory factors and an equilibrium of these factors (i.e. stimulatory and inhibitory) in the absence of immunosuppression is clearly required for normal EC growth and function. In HIV infection selective depletion of lymphocyte function occurs early in the disease (Dalgleish *et al.*, 1990). A number of studies have shown that the virus is able to interfere with antigen specific presentation (Manca *et al.*, 1990). Therefore in KS it is possible that lymphocyte subsets which exert a negative controlling influence on EC proliferation are deleted. Growth factors such as these produced by a variety of cells including the HTLV-II infected cell line described above could lead to KS. Moreover a variety of viruses and micro-organisms have been proposed as having a causative role in KS which only supports the contention that the stimulatory axis of KS need not be specific.

### HIV and the immune system

In addition to the 'global' immunosuppression engendered by the declining CD4 T helper cells, and the deletion of antigen specific subsets of T cells there is another poorly understood element which may affect the appearance of KS. HIV infected patients have non specifically activated immune systems as manifested by hypergammaglobulinaemia which is often restricted (oligoclonal) in quality (Habeshaw & Dalgleish, 1989). This is accompanied by elevated cytokines which may be stimulatory to EC cells. This non specific activation may be due to infected HIV cells expressing growth factors or it may be similar to the activation seen in chronic allogeneic disease or graft *vs* host disease (GVHD). It has been postulated that HIV could induce a GVH like disease by virtue of resembling a foreign MHC (Habeshaw *et al*., 1990). If this is so then immunological abnormalities associated with the dysregulation seen in GVHD (including the elevation of a number of cytokines) could further perturbate the immune system in a manner which could favour the development of KS.

### *Treatment of KS*

Treatment of KS depends upon the type and whether or not it is HIV related. Classical KS readily responds to minimal radiotherapy and chemotherapy (Odajnyk, 1985). However, it usually progresses so slowly that treatment is usually only given for cosmetic reasons. KS associated with HIV may also only require cosmetic treatment. Individual lesions may be unsightly even though they are neither aggressive of invasive. For instance the tip of the nose is a common site for KS and radiotherapy is of considerable cosmetic values. Systemic treatment often has to be considered in HIV infected individuals as lesions on the face and palate (another common site where RT may relieve unpleasant symptoms), often herald the involvement of the gastrointestinal tract and other visceral involvement. Apart from local treatment systemic treatment may be subdivided in (1) Anti-HIV treatment such as AZT (Zidovudine), (2) Systemic chemotherapy, (3) Immunomodulators such as interferon which also have antiviral properties.

KS in the present of HIV infection constitutes a diagnosis of AIDS which is usually, but not always, associated with advanced depletion of the immune system. Not surprisingly, AZT does not work well at this stage although AZT associated regression has been seen. Chemotherapy is limited by side effects to which AIDS patients are very vulnerable. In particular, chemotherapeutic neutropenia may be particularly ominous. Chemotherapeutic trials performed in Africa have suggested that vinblastine alone may be preferable to combination regimes such as Doxorubicin, bleomycin and vinblastine (ABV) because of the high incidence of opportunistic infections seen with the combination ABV (reviewed in Dalgleish, 1985). However a randomised trial in the US comparing ABV with etopiside (VP-16) and vinblastine as single agents showed a higher complete and partial response for ABV (84%) than etopiside and vinblastine alone (76% and 27%, respectively – although 50% had stable disease with vinblastine alone) (Lambeustein *et al*., 1989; Volberding *et al*., 1985). Chemotherpay may also be given intralesionally with some success. Interferon α (not β or α) has some effect on KS, but only at high doses (>36 million units) which suggests that it is acting as an antiproliferative agent. Although response rates of up to 40% have been reported, the doses required are associated with substantial toxicity. Combination studies of interferon with AZT and chemotherapy are in progress (Levine, 1990).

The increasing understanding of the basic biology of KS suggests that KS might be attacked either by eliminating the hosts response to the process of proliferation in endothelial cells, or by reducing the initial stimulus or by correcting the underlying immune defect. The latter is probably the most important in HIV infected patients as it is seen in transplantation patients where withdrawing immunosuppression is usually associated with complete regression. Even eliminating all HIV infection may not lead to any significant change in the immune response as the immune balance has been severely altered either indirectly or by clonal deletion of CD4 cells. In the meantime, careful study of KS with and without HIV infection is required for a better understanding of the aetiology of KS.

### Conclusions and future prospects

KS is not a one hit disease, but is the end product of a complex interplay between cellular proliferative and control systems which strongly invoke overall immunological control.

It is most serious in the presence of HIV infection and the greatest relevance in its treatment would be to eliminate HIV and reverse the associated change to the immune system. In the presence of HIV infection, treatment is complicated by the fact that it adds to the pre-existing immunosuppression. Treatment for KS in HIV negative patients is much easier and the biggest decision is when to treat.

Perhaps understanding the basic biology of KS at the cell and molecular level may throw new insights into the pathogenesis not only of KS, but also other proliferative diseases.

I would like to thank A. Kaul (London Hospital) for sharing valuable data and discussions which will be included in a forthcoming supplement of molecular aspects of medicine; AIDS.

### References

BAYLEY, A.C., DOWNING, R.G., CHEINGSONG-POPOV, R., TEDDER, R.S., DALGLEISH, A.G. & WEISS, R.A. (1985). HTLV-111 serology distinguishes atypical and endemic Kaposi's sarcoma in Africa. *Lancet*, **i**, 359.

BERAL, V., PETERMAN, T.A., BERKELMAN, R.L. & JAFFE, H.W. (1990). Kaposi's sarcoma among persons with AIDS: a sexually transmitted infection? *Lancet*, **i**, 123.

BUSSOLINO, F., WANG, J.M., DEFILIPPI, P. & 6 others (1989). Granulocyte- and granulocyte-macrophage- colony stimulating factors induce human endothelial cells to migrate and proliferate. *Nature*, **337**, 471.

DALGLEISH, A.G. (1985). Human retroviruses. *Aust. NZ J. Med*., **15**, 375.

DALGLEISH, A.G., HABESHAW, J. & MANCA, F. (1990). The pathogenesis of AIDS. In *AIDS and the New Viruses* Dalgleish, A.G. & Wiess, R.A. (eds). Academic Press: London.

DARROW, W.W., ECHENBERG, D.F., JAFFE, H.W. & 6 others (1987). Risk factors for human immunodeficiency virus infection in homosexual men. *Am. J. Publ. Health*, **77**, 479.

ENSOLI, B., NAKAMURA, S., SALAHUDDIN, S.Z. & 5 others (1989). AIDS-Kaposi's sarcoma-derived cells express cytokines with autocrine and paracrine growth effects. *Science*, **243**, 223.

FITZGERALD, O.M., HESS, E.V., CHANCE, A. & HIGHSMITH, R.F. (1987). Quantitative studies of human monokine-induced endothelial cell elongation. *J. Leukocyte Biol*., **41**, 421.

FOLKMAN, J. (1986). How is blood vessel growth regulated in normal and neoplastic tissues? *Can. Res*., **46**, 283.

FRIEDMAN-KIEN, A.E., SALTZMAN, B.R., LAO, Y. & 4 others (1990). Kaposi's sarcoma in HIV negative homosexual men. *Lancet*, **335**, 168.

GIRALDO, G., BETH, E. & HUANG, E.S. (1980). Kaposi's sarcoma and its relationship to cytomegalovirus (CMV). III. CMV DNA and CMV early antigens in Kaposi's sarcoma. *Int. J. Cancer*, **26**, 23.

GOEDERT, J.J., BIGGAR, R.J., MELBYE, M. & 8 others (1986). Effect of T4 count and cofactors on incidence of AIDS in homosexual men infected with human immunodeficiency virus. *JAMA*, **257**, 331.

GROENEWEGEN, G., BUURMAN, W.A. & VAN DER LINDEN, C.J. (1985). Lymphokines induce changes in morphology and enhance motility of endothelial cells. *Clin. Immunol. & Immunopath*., **36**, 378.

HABESHAW, J. & DALGLEISH, A.G. (1989). The relevance of HIV envelope/CD4 interactions to the pathogenesis of AIDS. *J. AIDS*, **2**, 457.

HABESHAW, J., DALGLEISH, A.G., BOUNTIFF, L. & 4 others (1990). AIDS pathogenesis; the role of cellular ligands in HIV infection. *Immunol. Today*, **11**, 618.

HARLAN, J.M. (1985). Leukocyte-endothelial interactions. *Blood*, **65**, 513.

HAVERKOS, H.W., PINSKY, P.F., DROTMAN, D.P. & BREGMAN, D.J. (1985). Disease manifestation among homosexual men with acquired immunodeficiency syndrome: a possible role of nitrates in Kaposi's sarcoma. *Sex Transm. Dis.*, **12,** 203.

HAVERKOS, H.W. (1987). Factors associated with the pathogenesis of AIDS. *J. Infect. Dis.*, **156,** 251.

LEVINE, A.M. (1990). Therapeutic approaches to neoplasms in AIDs. *Rev. Inf. Dis.*, **12,** 5.

LO, S.C. (1986). Isolation and identification of a novel virus from patients with AIDS. *Am. J. Trop. Med. Hyg.*, **35,** 675.

MAJEWSKI, S., TIGALONWA, M., JABLONSKA, S., POLAKOWSKI, I. & JANCZURA, E. (1987). Serum samples from patients with active psoriasis enhance lymphocyte induced angiogenesis and modulate endothelial cell proliferation. *Arch. Dermatol.*, **123,** 221.

MANCA, F., HABESHAW, J. & DALGLEISH, A.G. (1990). HIV envelope glycoprotein antigen specific T cell responses and soluble CD4. *Lancet*, **i,** 811.

MANN, D.L., MURRAY, C., O'DONNELL, M., BLATTNER, W.A. & GOEDERT, J.J. (1990). HLA Antigen frequencies in HIV-1 related Kaposi's sarcoma. *J. AIDS*, **1** (Suppl), 551.

MATHUR-WAGH, U., MILDVAN, D. & SENIE, R.T. (1985). Follow-up at 4 1/2 years on homosexual men with generalised lymphadenopathy. *N. Engl. J. Med.*, **313,** 1542.

NAKAMURA, S., SALAHUDDIN, S.Z. & BIBERFELD, P. & 4 others (1988). Kaposi's sarcoma cells: long-term culture with growth factor from retrovirus-infected CD4+ T cells. *Science*, **242,** 426.

OSMOND, D., MOSS, A.R., BACHETTI, P., VOLBERDING, P., BARRIE-SINOUSSI, F. & CHERMAN, J.E. (1985). A case-control study of risk factors for AIDS in San Francisco. Presented at the *1st International Conference on Acquired Immunodeficiency Syndrome (AIDS)*, Atlanta, April 14–17.

PAPASTERIADES, C., KALOTERAKIS, A., FILIOTUO, A. & 5 others (1984). Histocompatibility antigens HLA-A, B, DR in Greek patients with Kaposi's sarcoma. *Tissue Antigens*, **24,** 313.

PENN, I. (1979). Kaposi's sarcoma in organ transplant recipients. Report of 20 cases. *Transplanation*, **27,** 8.

PENN, I. (1983). Kaposi's sarcoma in immunosuppressed patients. *J. Clin. Lab. Immunol.*, **11,** 47.

POBER, J.S. (1988). Cytokine-mediated activation of vascular endothelium. *Am. J. Pathol.*, **133,** 426.

POLK, F., FOX, R., BROOKMEYER, R. & 5 others (1987). Predictors of the acquired immunodeficiency syndrome developing in a cohort of seropositive men. *N. Eng. J. Med.*, **316,** 62.

POLLACK, M.S., SAFAI, B., MYSKOWSKI, P.L., GOLD, J.W.M., PANDEY, J. & DUPONT, B. (1983*a*). Frequencies of HLA and Gm immunogenetic markers in Kaposi's sarcoma. *Tissue Antigens*, **21,** 1.

POLLACK, M.S., SAFAI, B. & DUPONT, B. (1983*b*). HLA-DR5 and DR2 susceptibility factors for acquired immunodeficiency syndrome patients with Kaposi's sarcoma in different ethnic subpopulations. *Dis. Markers*, **1,** 135.

POLLACK, M.S. & RICH, R.R. (1985). The HLA complex and the pathogenesis of infectious disease. *J. Infect. Dis.*, **151,** 1.

RAPPERSBERGER, K., TSCHALCHLER, E., ZONZITS, E. & 10 others (1990). Endemic KS in HIV-1 negative persons demonstration of retrovirus like particles in cutaneous lesions. *J. Invest. Dermatol.*, **95,** 371.

RUTHERFORD, G.W., PAYNE, S.F. & LEMP, G.F. (1990). The epidemiology of AIDS related Kaposi's sarcoma in San Francisco. *J. AIDS*, **3** (Suppl), 54.

SALAHUDDIN, S.Z., NAKAMURA, S., BIBERFIELD, P. & 4 others (1988). Angiogenic properties of Kaposi's sarcoma-derived cells after long-term, culture *in vitro*. *Science*, **242,** 430.

SERWADDA, D., MUGERWA, R.D., SEWANKAMBO, N.K. & 9 others (1985). Slim disease: a new disease in Uganda and its association with HTLV-111 infection. *Lancet*, **ii,** 849.

STREETER, P.R., BERG, E.L., ROUSE, N., BORGATZE, R. & BUTCHER, E.C. (1988). A tissue specific endothelial cell molecule involved in lymphocyte homing. *Nature*, **331,** 41.

TEMPLETON, A.C. (1973). (ed.) *Tumours in a Tropical Country*. Springer Verlag: Berlin.

VOGEL, J., HINRICHS, S.H., REYNOLDS, R.K., LUCISIS, P. & JAY, G. (1988). The HIV tat gene induces dermal lesions resembling Kaposi's sarcoma in transgenic mice. *Nature*, **335,** 606.

ZEIGLER, J.L. & DORFMAN, R.F. (1988). (eds) *Kaposi's Sarcoma*. Marcel Dekker: New York.


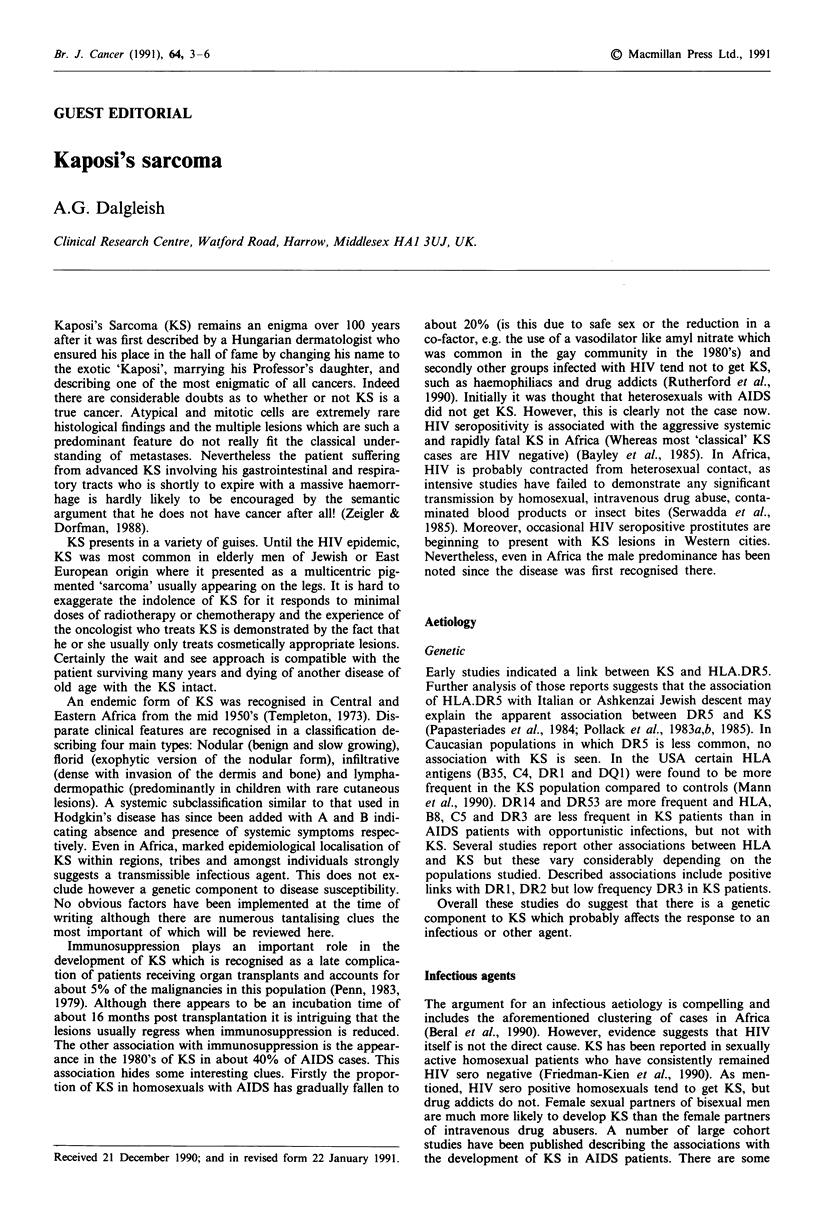

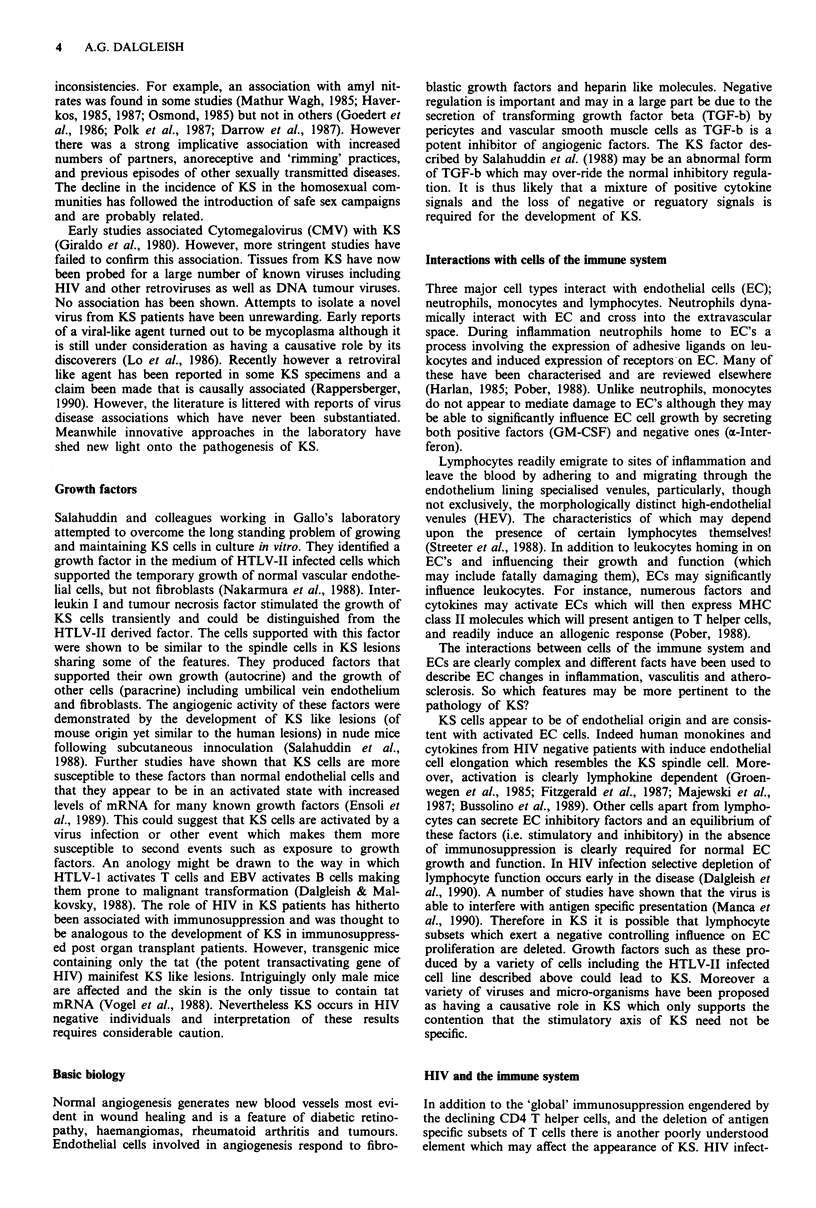

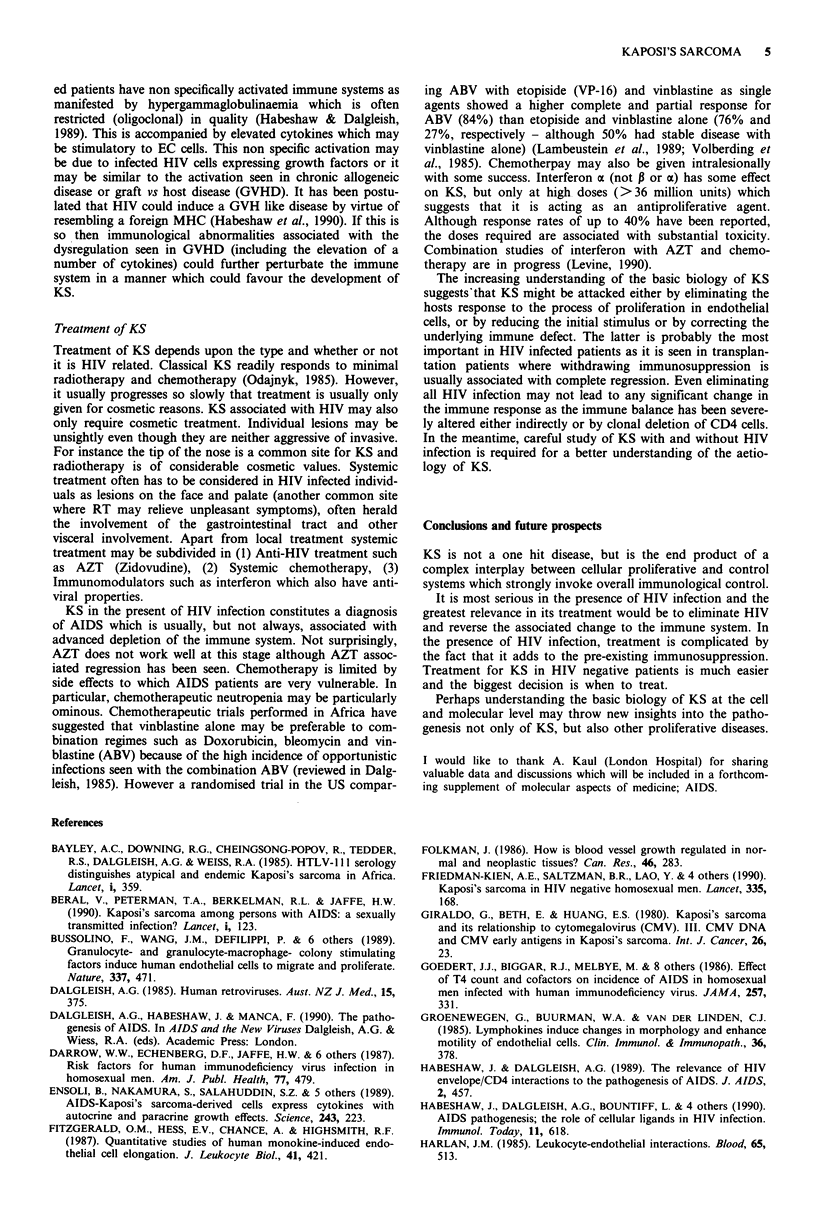

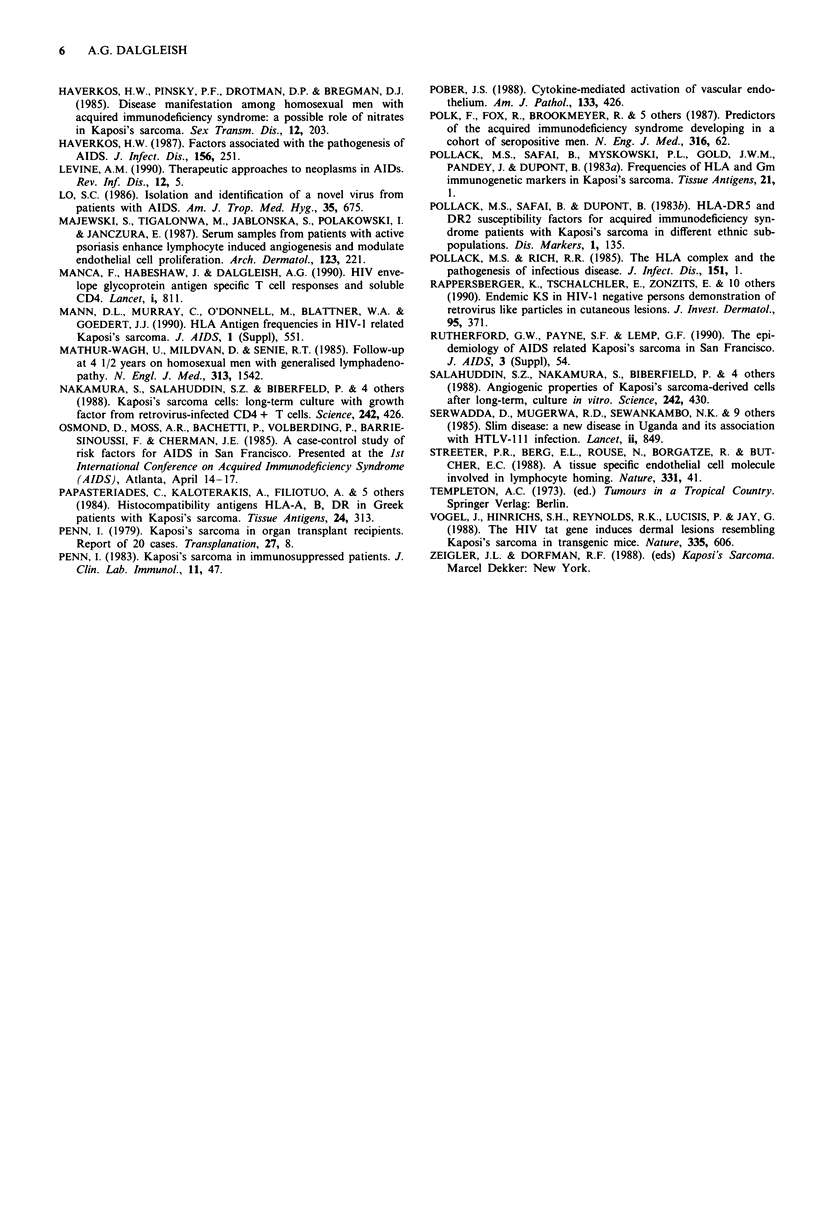


## References

[OCR_00394] Bayley A. C., Downing R. G., Cheingsong-Popov R., Tedder R. S., Dalgleish A. G., Weiss R. A. (1985). HTLV-III serology distinguishes atypical and endemic Kaposi's sarcoma in Africa.. Lancet.

[OCR_00400] Beral V., Peterman T. A., Berkelman R. L., Jaffe H. W. (1990). Kaposi's sarcoma among persons with AIDS: a sexually transmitted infection?. Lancet.

[OCR_00405] Bussolino F., Wang J. M., Defilippi P., Turrini F., Sanavio F., Edgell C. J., Aglietta M., Arese P., Mantovani A. (1989). Granulocyte- and granulocyte-macrophage-colony stimulating factors induce human endothelial cells to migrate and proliferate.. Nature.

[OCR_00411] Dalgleish A. G. (1985). Human retroviruses.. Aust N Z J Med.

[OCR_00420] Darrow W. W., Echenberg D. F., Jaffe H. W., O'Malley P. M., Byers R. H., Getchell J. P., Curran J. W. (1987). Risk factors for human immunodeficiency virus (HIV) infections in homosexual men.. Am J Public Health.

[OCR_00425] Ensoli B., Nakamura S., Salahuddin S. Z., Biberfeld P., Larsson L., Beaver B., Wong-Staal F., Gallo R. C. (1989). AIDS-Kaposi's sarcoma-derived cells express cytokines with autocrine and paracrine growth effects.. Science.

[OCR_00430] FitzGerald O. M., Hess E. V., Chance A., Highsmith R. F. (1987). Quantitative studies of human monokine-induced endothelial cell elongation.. J Leukoc Biol.

[OCR_00439] Friedman-Kien A. E., Saltzman B. R., Cao Y. Z., Nestor M. S., Mirabile M., Li J. J., Peterman T. A. (1990). Kaposi's sarcoma in HIV-negative homosexual men.. Lancet.

[OCR_00444] Giraldo G., Beth E., Huang E. S. (1980). Kaposi's sarcoma and its relationship to cytomegalovirus (CMNV). III. CMV DNA and CMV early antigens in Kaposi's sarcoma.. Int J Cancer.

[OCR_00450] Goedert J. J., Biggar R. J., Melbye M., Mann D. L., Wilson S., Gail M. H., Grossman R. J., DiGioia R. A., Sanchez W. C., Weiss S. H. (1987). Effect of T4 count and cofactors on the incidence of AIDS in homosexual men infected with human immunodeficiency virus.. JAMA.

[OCR_00456] Groenewegen G., Buurman W. A., van der Linden C. J. (1985). Lymphokines induce changes in morphology and enhance motility of endothelial cells.. Clin Immunol Immunopathol.

[OCR_00462] Habeshaw J. A., Dalgleish A. G. (1989). The relevance of HIV env/CD4 interactions to the pathogenesis of acquired immune deficiency syndrome.. J Acquir Immune Defic Syndr.

[OCR_00472] Harlan J. M. (1985). Leukocyte-endothelial interactions.. Blood.

[OCR_00484] Haverkos H. W. (1987). Factors associated with the pathogenesis of AIDS.. J Infect Dis.

[OCR_00478] Haverkos H. W., Pinsky P. F., Drotman D. P., Bregman D. J. (1985). Disease manifestation among homosexual men with acquired immunodeficiency syndrome: a possible role of nitrites in Kaposi's sarcoma.. Sex Transm Dis.

[OCR_00492] Lo S. C. (1986). Isolation and identification of a novel virus from patients with AIDS.. Am J Trop Med Hyg.

[OCR_00496] Majewski S., Tigalonowa M., Jabłnska S., Polakowski I., Janczura E. (1987). Serum samples from patients with active psoriasis enhance lymphocyte-induced angiogenesis and modulate endothelial cell proliferation.. Arch Dermatol.

[OCR_00502] Manca F., Habeshaw J. A., Dalgleish A. G. (1990). HIV envelope glycoprotein, antigen specific T-cell responses, and soluble CD4.. Lancet.

[OCR_00512] Mathur-Wagh U., Mildvan D., Senie R. T. (1985). Follow-up at 41/2 years on homosexual men with generalized lymphadenopathy.. N Engl J Med.

[OCR_00517] Nakamura S., Salahuddin S. Z., Biberfeld P., Ensoli B., Markham P. D., Wong-Staal F., Gallo R. C. (1988). Kaposi's sarcoma cells: long-term culture with growth factor from retrovirus-infected CD4+ T cells.. Science.

[OCR_00528] Papasteriades C., Kaloterakis A., Filiotou A., Economidou J., Nicolis G., Trichopoulos D., Stratigos J. (1984). Histocompatibility antigens HLA-A, -B, -DR in Greek patients with Kaposi's sarcoma.. Tissue Antigens.

[OCR_00533] Penn I. (1979). Kaposi's sarcoma in organ transplant recipients: report of 20 cases.. Transplantation.

[OCR_00537] Pereira H. A., Shelton M. J., Hosking C. S. (1983). Neutrophil iodination micro-method as an index of neutrophil and opsonic function.. J Clin Lab Immunol.

[OCR_00541] Pober J. S. (1988). Warner-Lambert/Parke-Davis award lecture. Cytokine-mediated activation of vascular endothelium. Physiology and pathology.. Am J Pathol.

[OCR_00562] Pollack M. S., Rich R. R. (1985). The HLA complex and the pathogenesis of infectious diseases.. J Infect Dis.

[OCR_00550] Pollack M. S., Safai B., Myskowski P. L., Gold J. W., Pandey J., Dupont B. (1983). Frequencies of HLA and Gm immunogenetic markers in Kaposi's sarcoma.. Tissue Antigens.

[OCR_00566] Rappersberger K., Tschachler E., Zonzits E., Gillitzer R., Hatzakis A., Kaloterakis A., Mann D. L., Popow-Kraupp T., Biggar R. J., Berger R. (1990). Endemic Kaposi's sarcoma in human immunodeficiency virus type 1-seronegative persons: demonstration of retrovirus-like particles in cutaneous lesions.. J Invest Dermatol.

[OCR_00577] Salahuddin S. Z., Nakamura S., Biberfeld P., Kaplan M. H., Markham P. D., Larsson L., Gallo R. C. (1988). Angiogenic properties of Kaposi's sarcoma-derived cells after long-term culture in vitro.. Science.

[OCR_00582] Serwadda D., Mugerwa R. D., Sewankambo N. K., Lwegaba A., Carswell J. W., Kirya G. B., Bayley A. C., Downing R. G., Tedder R. S., Clayden S. A. (1985). Slim disease: a new disease in Uganda and its association with HTLV-III infection.. Lancet.

[OCR_00589] Streeter P. R., Berg E. L., Rouse B. T., Bargatze R. F., Butcher E. C. (1988). A tissue-specific endothelial cell molecule involved in lymphocyte homing.. Nature.

[OCR_00596] Vogel J., Hinrichs S. H., Reynolds R. K., Luciw P. A., Jay G. (1988). The HIV tat gene induces dermal lesions resembling Kaposi's sarcoma in transgenic mice.. Nature.

